# First report of hypoplastic left heart syndrome in 3p- syndrome and review of candidate genes

**DOI:** 10.1590/1984-0462/2025/43/2024133

**Published:** 2025-01-20

**Authors:** Ana Kalise Böttcher, Monique Banik Siqueira, Natasha Malgarezi, Marcela Rodrigues Nunes, Rafaella Mergener, Luisa Pigatto Kalil, Patrícia Trevisan, Paulo Ricardo Gazzola Zen

**Affiliations:** a Universidade Federal de Ciências da Saúde de Porto Alegre, Porto Alegre, RS, Brazil.; b Universidade do Vale do Rio dos Sinos, São Leopoldo, RS, Brazil.; c Irmandade da Santa Casa de Misericórdia de Porto Alegre, Porto Alegre, RS, Brazil.; d University of Colorado Anschutz Medical Campus, Aurora, CO, United States of America.

**Keywords:** Cardiomyopathies, Heart defects, congenital, Hypoplastic left heart syndrome, Chromosome disorders, Cytogenetic analysis, Fetal heart, Genetic diseases; inborn., Cardiomiopatias, Cardiopatias congênitas, Transtornos cromossômicos, Análise citogenética, Coração fetal, Doenças genéticas inatas.

## Abstract

**Objective:**

3p deletion syndrome is a rare monosomal disease that encompasses deletions throughout the short arm of chromosome 3. It is often in the distal region (3p25-pter), but variations in breakpoints and a complex clinical manifestation exist, with congenital heart defects being considered rare. We present the first case of hypoplastic left heart syndrome and minor dysmorphic features associated with 3p- syndrome. Furthermore, we aim to establish a gene-phenotype association.

**Case description:**

The diagnosis was made by karyotyping, followed by a literature investigation and *in silico* bioinformatic analysis about the possible candidate genes associated with congenital heart defects or hypoplastic left heart syndrome in 3p- syndrome. All genes analyzed that could affect heart formation are located in the 3p25.3 region, adjacent to the deleted region in the newborn from our case (3p26). Taking into account the technical limitations of the karyotype and the strength of evidence from each gene evaluated and locus proximity, it is likely that an unidentified partial break in the *CAV3* gene occurred.

**Comments:**

We identified an indirect relation between gene *CAV3* and hypoplastic left heart syndrome due to its strong association with cardiomyopathies and isolated cardiac defects. Furthermore, the cytogenetic band from our case is new information for the delimitation of a critical cardiac region on 3p syndrome, a discussion that has been ongoing since 1986. Thus, we reinforce the importance of cytogenetic investigation in patients with hypoplastic hearts and dysmorphia, assisting in diagnosis, definition of prognosis, and genetic counseling for the family.

## RESUMO

**Objetivo:**

A síndrome da deleção 3p é uma doença monossômica rara que engloba deleções em todo o braço curto do cromossomo 3. Frequentemente está na região distal (3p25-pter), mas apresenta variações nos pontos de quebra e uma manifestação clínica complexa, cujas cardiopatias congênitas são consideradas raras. Apresentamos aqui o primeiro caso de síndrome do coração esquerdo hipoplásico e características dismórficas menores na síndrome 3p-. Além disso, buscamos estabelecer uma associação gene-fenótipo.

**Descrição do caso:**

O diagnóstico foi feito por cariótipo e procedeu-se a uma investigação bibliográfica e análise bioinformática in silico sobre os possíveis genes associados a cardiopatias congênitas ou síndrome do coração esquerdo hipoplásico na síndrome da deleção 3p. Todos os genes analisados que poderiam afetar a formação do coração estão localizados na região 3p25.3, adjacente à região deletada no recém-nascido do nosso caso (3p26). Tendo em conta as limitações técnicas do cariótipo, a força da evidência de cada gene avaliado e a proximidade do locus, é provável que tenha ocorrido uma quebra parcial não identificada no gene CAV3.

**Comentários:**

Identificamos uma relação indireta entre o gene CAV3 e a síndrome do coração hipoplásico esquerdo, em razão da sua forte associação com cardiomiopatias e defeitos cardíacos isolados. Além disso, a banda citogenética do nosso caso é uma informação nova para a delimitação de uma região cardíaca crítica na síndrome 3p, discussão que está em andamento desde 1986. Assim, reforçamos a importância da investigação citogenética em pacientes com coração hipoplásico e dismorfias auxiliando no diagnóstico, definição de prognóstico e aconselhamento genético para a família.

## INTRODUCTION

Distal deletion of chromosome 3p25-pter (3p- syndrome) (OMIM #613792) is a rare monosomal disorder with fewer than 60 reported cases worldwide.^1^ Both terminal and middle deletions in the short arm of chromosome 3 may cause the syndrome; however, losses in the terminal part are the most common.^1^

It produces a distinct clinical syndrome characterized by hypotonia, low birth weight, delayed neurological and motor development (crawling and walking), speech delay, intellectual impairment, telecanthus, craniofacial dysmorphia (microcephaly, micrognathia, and ptosis), and congenital heart defects (CHDs).^2^ The latter are rare manifestations, with atrioventricular septal defect being the most common among them.^1,2^

Hypoplastic left heart syndrome (HLHS) is a severe heart defect responsible for 1–3.8% of all CHDs.^3^ It is characterized by the underdevelopment of the left side of the heart, including the left ventricle morphological underdevelopment, associated with atresia or stenosis of the mitral valves, and aortic hypoplasia.^3^ This condition causes heart failure as the blood is not pumped adequately to the body.^3^ The right ventricle compensates along with the persistence of the arterial duct and foramen ovale.^3^ However, once it closes, administration of prostaglandins and surgical intervention become necessary.^3^ HLHS is responsible for 23% of deaths during the neonatal period.^3^

HLHS can be diagnosed prenatally, starting from 18 weeks, through fetal echocardiography.^3,4^ The etiology of this condition is still unclear, but it is estimated that 25% of affected fetuses have an associated genetic disease or a major malformation.^4^ It has a profound genetic heterogeneity and oligogenic etiology, in which several chromosomal alterations, such as Jacobsen syndrome, Turner syndrome, and trisomies 13, 18, and 9, have been identified.^5^ Therefore, once the condition is diagnosed through a fetal echocardiography, additional evaluations are recommended, including genetic screening and the exclusion of extracardiac anomalies.^3^

The aim of this case report is to present, for the first time, the diagnostics of HLHS in a newborn (NB) with karyotype 46,XX,del(3)(p26). In addition, we discuss the possibility of a phenotype amplification for 3p deletion syndrome and the genes already associated with cardiac defects that could be involved in the development of this pathology. Therein, the literature review covered genes present in the 3p region associated with cardiopathies or isolated cardiac defects, or more specifically HLHS.

## METHOD

Clinical and laboratory data were collected from the patient’s medical records. The study was approved by the Institutional Ethics and Research Committee (CAAE:74971917.2.0000.5683).

The bioinformatic analysis was performed based on the patient’s karyotype, using the ClinGen, DECIPHER, and OMIM programs. The results obtained in DECIPHER were filtered using the pHaplo score. It reflects the haploinsufficiency potential of a gene, that is, how likely the loss of gene function would be when one of the copies is deleted, indicating that only one wild copy of that gene would not be enough to maintain its function. In this, the genes are listed from 0.0 to 1, with 0.0 being the least likely to lose its function with the deletion of one of the copies and 1 being the most likely. Only genes that presented a pHaplo value of ≥ 0.5 had their functions analyzed in OMIM, trying to find a correlation with the patient’s phenotype.^6,7^ An additional investigation on the OMIM platform with the descriptor “cardiomyopathy” happened to increase possible genes located in chromosome 3p25-pter involved in cardiac development (Table 1).

**Table 1:** Genes associated with cardiomyopathies by OMIM.

Gene	Cytogenetic location
CAV3	3p25.3
CMD1NN	3p25.2
RAF	3p25.2
ARVD5	3p25.1

The literature review included 14 articles. From these, four articles were filtered by the terms “Chromosome 3,” “Cardiopathies,” and “Hypoplastic Left Heart Syndrome” anywhere in the text within the NCBI or Google Scholar databases.^1,8,9,10^ The following filters were applied: publication in the last 10 years; language English or Portuguese. Exclusion criteria were publications in annals or congresses, and alteration in other parts of the genome and nonhuman species. The choice was based on reading the titles and abstracts. Active reading from the references included nine other articles.^11,12,13,14,15,16,17,18,19^

For posterior analysis of the gene of interest, a secondary narrative review was performed, with the descriptors “CAV3”, “Hypoplastic Left Heart Syndrome”, “Cardiomyopathy”, and/or “Myocardium” in the same terms as before. Articles addressing histology, genetics, medical cardiology, anatomy, or physiology were included.

## RESULTS

The patient was the first-born daughter of a young, healthy, and non-consanguineous couple. The mother presented high blood pressure and urinary tract infection three times during gestation. She denied the use of drugs, tobacco, and alcohol. Prenatal ultrasound revealed a cervical cystic hygroma measuring 4.1 x 2.5 cm, and an echocardiogram indicated severe hypoplasia of the mitral and aortic valves, left ventricular hypoplasia, a small ventricular septal defect, and a hypoplastic aortic arch. The G-banding karyotype analysis identified a 3p26 deletion [46,XX,del(3)(p26)].

**Figure 1:**
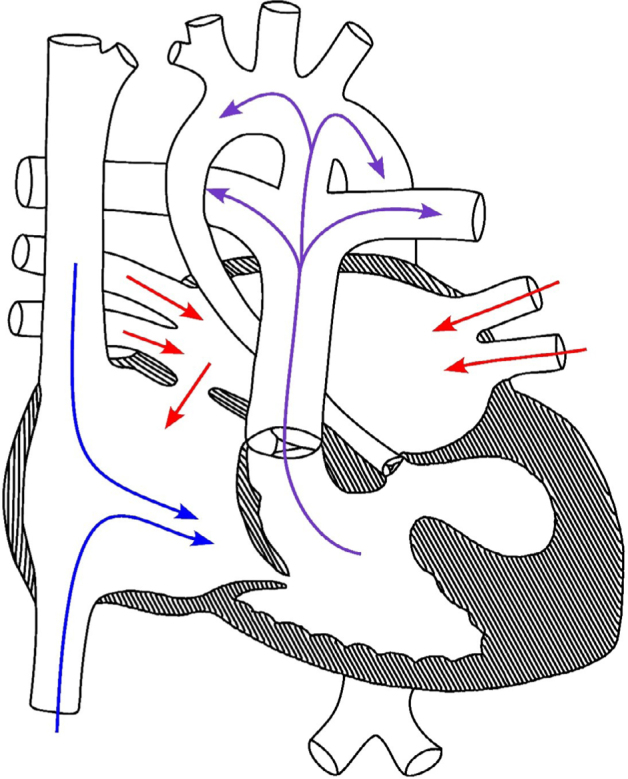
Hemodynamic of hypoplastic left heart syndrome MA/AA hearts. The hypoplastic left heart syndrome of our patient is characterized by underdeveloped left heart structures, including a diminutive left ventricle, with mitral and aortic valve atresia, ventricular septal defect, and aortic isthmus hypoplasia. The left ventricle has little strength to bump the blood during systole and no way of receiving arterial blood from left atrium, which leads to a systolic insufficiency. To overpower that, arterial blood bypasses the left ventricle by following through the patent foramen ovale, from the left atrium to the right atrium, and then coming through normal circulation until the pulmonary artery. From there, it takes the patent arterial duct connection with the aorta, enabling it to be distributed to the rest of the body. Because of patent foramen ovale, the baby presents with cyanosis. The pulmonary artery dilates in response to the extra volume (systolic volume) it receives. The right ventricle compensates for the preload and afterload of the nonfunctional ventricle by contracting vigorously to supply the body. In this scenario, right ventricle volume is force filled, leading to elevated internal pressure, enlargement of the right ventricle cavity dimensions, and the development of mild diastolic dysfunction. Also, the aorta is inhibited by the development of right-side structures, presenting itself thinner than usual.

Source: Created by the authors using Inkscape 1.3.0, Porto Alegre, RS, Brazil.

The patient was delivered via cesarean section at 38 weeks and 6 days of gestational age, with birthweight 2842 kg (P8th), height 42 cm (<P0,4th), and cephalic perimeter measured 34 cm (P45th). The Apgar score was 6/8. The NB required reanimation and assisted ventilation. An echocardiogram performed after birth confirmed a diagnosis of HLHS, with mitral and aortic valve atresia and a ventricular septal defect. Surgical intervention was performed when the patient was 5 days old. The hemodynamic pattern is represented in Figure 1. Brain and abdominal ultrasounds showed no abnormalities. On dysmorphological examination, loose skin on the neck, a wide anterior fontanelle, a short nose, a micrognathia-like impression, clinodactyly of the fifth finger on both hands (indicative of medial phalangeal hypoplasia), and a second toe-like hallux in both feet were observed. The patient died at 6 days old. The parents declined to proceed with the investigations, which made it impossible to investigate the origin of the genetic alteration and to provide family counseling.

**Figure 2:**
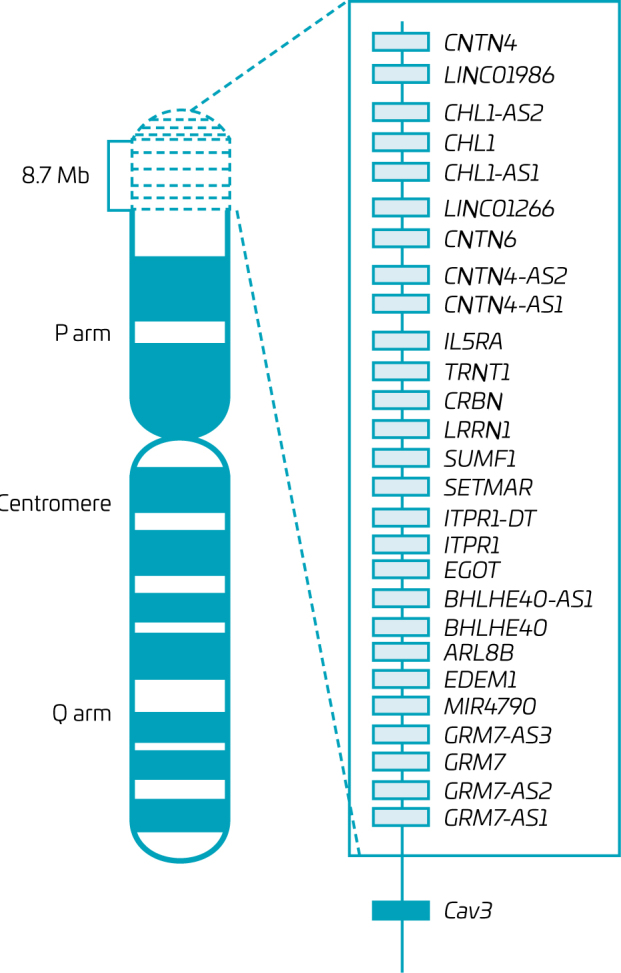
Representation of the patient chromosome 3p26 deletion. The deleted genes are listed in the box, according to the locus order. Our target gene (CAV3) is represented outside the box in its respective distance from the deleted region.

Source: Created by the authors using BioRender.com (2023), Porto Alegre, RS, Brazil.

By bioinformatic and molecular analysis, we identified that the deleted region, 3p26.1 to 3p26.3, covers approximately 8,700,000bp (UCSC Genome Browser on Human GRCh38/hg38). The ClinGen program identified 27 genes (Figure 2) in this region, of which 15 are protein-coding genes (DECIPHER). After haploinsufficiency analysis, 12 genes presented a value of ≥0.5, mostly correlated with neurological disorders, metabolic alterations, and delays in mental and intellectual development. None of the genes in the study region could be correlated with the patient’s heart condition, neither directly nor indirectly.

## DISCUSSION

Until now, the HLHS had only been described in a 3p chromosome deletion in association with the *FOXP1* gene on band 3p13.^8^ Based on this bioinformatic analysis and literature investigation, we could not find a gene in the 3p26 region correlated with HLHS, which suggests some microdeletion in another region of the patient genome, not evident in the karyotype analysis. As for other cardiac problems associated with 3p- syndrome, including deletion cases encompassing the 3p26 region, only isolated heart defects have been reported.

The first report was from Tolmie et al. in 1986, who described a patient with a ventricular septal defect and karyotype [46, XY, del(3)(p25pter)].^11^ Later, Ramer et al. in 1989 reported a patient with a complete endocardial cushion defect and moderate congestive heart failure, clinically managed, with [46, XY, del(3)(p25pter)].^12^ Both cell lines were used in subsequent studies of other authors. The lymphoblastic cell line GM10922, corresponding to Ramier’s patient sample, was stored at NIGMS (Human Genetic Mutant Cell Repository Coriell Institute for Medical Research, Camden, NJ). We have not located the identification of Tolmie’s patient lineage – referred to as GMTPL from here.

Phipps et al. in 1994 performed Fluorescence in Situ Hybridization (FISH) on GM10922, GMTPL, a new case [46, XX, del(3)(p25p26)] with an atrial septal defect (sample CUMG3.4) and two more carriers of the 3p- syndrome without CHDs.^13^ Based on the comparison, genetic regions from range D3S1250 to D3S18 were suggested as involved with cardiac development (Table 2).^13,14,16^ Isolating the *G6, VHL*, and *PMCA2 (ATP2B2)* genes, they contemplate only the latter as a candidate for septal defects.^13^

**Table 2:** Investigated markers in patients with congenital heart disease.^13,14,16^

Green et al.^16^	3p25										
Markers	GM10922	GMTPL	CUMG3.4	P1	P4						
D3S1585	+	+	+	+	+						
D3S3610	+	+	+	+	+						
D3S3693	+	+	+	+	+	Drumheller et al.^14^					
D3S3602	-	+	+	-	+	Markers	GM10922				
D3S3701	NR	+	+	NR	+	D3S1259	+	Phipps et al.^13^	GM10922	GMTPL	CUMG3.4
D3S1259	-	-	+	-	+	D3S1252	+	Markers			
D3S3088	-	-	+	-	+	D3S1255	+	D3S651	NR	+	+
D3S3680/D3S3714	NR	-	+	NR	+	D3S1110	+	RAF1	+	+	+
NIB1677	-	-	+	-	+	**D3S1585**	+	D3S732	NR	-	+
**D3S1263**	-	-	+	-	+	**D3S1263**	-	**D3S125O**	-	-	+
**D3S3594**	-	NR	-	-	-						
**D3S587**	-	-	-	-	-	**D3S587**	-	**D3S587**	-	-	-
**D3S3589**	NR	-	-	NR	-						
**D3S1038**	-	-	-	-	-	**D3S1038**	-	**D3S1038**	NR	NR	-
**D3S601**	-	NR	-	-	-	**D3S601**	-	**D3S601**	-	-	
**D3S1317**	NR	NR	NR	NR	NR	**D3S1317**	-	**D3S1317**	NR	NR	NR
						VHL3’	-				
						VHL5’	-				
						223 bp STS	-				
D3S1597	NR	-	NR	NR	NR	D3S1597	-				
D3S18	NR	-	-	NR	-	D3S18	-	**D3S18**	-	-	-
D3S1304	NR	NR	NR	NR	NR			D3S1442	-	-	-
								D3S1443	-	-	-
								D3S1444	-	-	-
3pter											

Note that not all markers are aligned, but they are ordered according to chromosome direction (3p25⟶3pter) and at right are the oldest studies. Regions considered critical by the author are in red. NR: Not rated/informed.

Drumheller et al. in 1996 investigated FISH GM10922 in comparison to other patients with 3p- syndrome without CHDs.^14^ They applied different markers (Table 2), as well as a Polymerase Chain Reaction (PCR). It was proposed to restrict the gene region considered critical for CHDs to markers D3S1585 to D3S1317 since this region was deleted only in patients with CHD.^14^ Within the new range, *SEC13R* was highlighted and *PMCA2* was reinforced, as previously suggested by Phipps et al.^13^

Green et al. performed FISH considering other microsatellite markers (Table 2) and PCR.^16^ They included GM10922, GMTPL, CUMG3.4, and two new patients with CHDs (unspecified, called Patient 1, P1, and Patient 4, P4). The gene region proposed was in the range of D3S1263–D3S3594.^16^ It comprises the *SLC6A11*, *SLC6A1*, *HRH1*, and *ATG7* genes, but excludes other possible CHD candidates suggested before, such as *PMCA2, TIMP4,* and *SEC13R*.^16^ Thus, assuming gene non-penetrance in cases without CHDs, the authors concluded that it would be likely that the haploinsufficiency of one or more genes in the critical interval could cause failures in cardiac development. Yet, two patients compared in the authors’ analysis had the D3S3594 region deleted and did not present CHD. Therefore, the proposed region is not appropriate. Malmgren et al. also evaluated, by the same methods, two new cases of 3p- deletion without CHDs and reassessed GM10985.^17^ One of the new patients was found to have an *SLC6A11* deletion without developing CHDs. With this, the authors propose restricting more of the critical region previously suggested by Green et al.^16^ excluding the *SLC6A11* gene. Of the three candidate genes that remained in the interval (*SLC6A1*, *HRH1*, and *ATG7)*, none has a known action on cardiac development.

With modern techniques, Shuib et al. reunited 16 patients (5 presenting CHD) with cytogenetically detectable deletions of 3p25 for investigation by Single Nucleotide Polymorphism Microarray (SNP-Array) and Multiplex Ligation-dependent Probe Amplification (MLPA).^18^ They mapped the candidate site for CHDs as approximately 200 kb, assuming complete penetrance.^18^ This interval contains only the *HRH1* and *ATG7* genes but again, none of these were directly linked to heart disease.^18^ The authors highlight that CRELD1 gene, which has been suggested as a 3p25 AVSD, maps outside the 3p- CHD target intervals and are sporadic.

Gunnarsson et al. reported a patient [46, XX, del(3)(p25.3-p26.1)] evaluated by SNP-Array and confirmed through MLPA.^19^ She had an atrial and septal valve defect, a large coronary sinus (suggestive of an anomaly in the return of the venous system), and presented with a horizontal membrane, which divides the atrium (suggestive of cor triatriatum sinistrum). None of the genes in the interstitial deleted portion coincide with the critical regions proposed. In agreement with the thought of Sotgia et al.,^15^ Gunnarson et al.^19^ stated that the haploinsufficiency of the *CAV3* gene is the most likely cause of CHDs on 3p- syndrome.^15,19^ This gene encodes caveolin-3, a membrane protein that makes up caveolae and is expressed in all three muscle types.^15^ Mutations in this cause clinically heterogeneous neuromuscular disturbances or heart disorders.^15,20^ It encompasses the D3S4539, D3S4163, and D3S18 markers and is close to the oxytocin receptor 3’ end, with D3S18 being associated with von Hippel–Lindau disease and the 3p- syndrome itself.^20^ Zhang et al., for example, described a patient [46,XX,r(3)(p25.3q29)] with patent ductus arteriosus, patent foramen ovale, and an atrial septal defect.^9^ The breakpoints revealed a 10Mb deletion in 3p25.3 that resulted in the loss of 42 genes, including *CAV3*, the only gene among those pointed out by this review.^9^ Finally, Fu et al. in 2021 reported a case [46,XY,del(3)(p25.3p26.3)], diagnosed by array, whose patient had CHD (unspecified), supporting *SEC13R*, *SLC6A11,* and *CAV3* genes as candidates.^1^

The location of all candidate genes for the cardiac aspects registered in 3p syndrome as proposed by the authors is shown in Table 3. However, none correspond to the excluded portion of the present case (3p26). Coincidentally, all analyzed genes that could affect heart formation (Tables 1 and 3) are located in the 3p25.3 region, adjacent to the deleted region in the NB in our case. As known, an investigation by G-banded karyotype is the gold standard for detecting large deletions, secondary deletions, duplications, inversions, or translocations through band pairing. Nevertheless, alterations smaller than 5Mb are difficult to visualize, making it impossible to identify the involved genes. The genetic team advised performing high-resolution karyotyping, which would allow superior band visibility, including identification of microdeletions and breakpoint locations, thus providing greater precision regarding the deleted region. Unfortunately, the NB died before further examinations could be conducted and there was not enough biological material stored of her, preventing us from running additional and more complete genetic tests, such as microarray-based comparative genomic hybridization (CGH-Array). Therefore, it became impossible to confirm the breakpoint and our research proceeded with important limitations.

**Table 3:** Cytogenetics location of the genes proposed in the literature review as candidates for heart disease.

Gene	Cytogenetic location	Genomic coordinates (GRCh38)
CAV3	3p25.3	3:8,733,802–8,746,758
CRELD1	3p25.3	3:9,933,834–9,945,406
VHL	3p25.3	3:10,141,778–10,153,667
SEC13R	3p25.3	3:10,300,931–10,321,112
PMCA2	3p25.3	3:10,324,023–10,708,007
SLC6A11	3p25.3	3:10,816,228–10,940,714
SLC6A1	3p25.3	3:10,992,748–11,039,247
HRH1	3p25.3	3:11,137,238–11,263,557
ATG7	3p25.3	3:11,272,397–11,576,353

So far, there is no conclusive evidence regarding which gene in 3p syndrome could have caused the HLHS as this is merely the first reported case. However, the limitations of the diagnostic method used in our case, combined with the data available in the literature, allow for hypothesizing an unidentified partial breakpoint reaching 3p25.3 and one of the candidate genes for CHDs discussed. Given that, the *CAV3* locus is the nearest to our patient deletion site (Figure 2), with only two genes (*LMCD1* and *SSUH2*) in between that lack clinical correlation. Specifically, *CAV3* is of interest as it has been consistently associated with CHDs in reports that utilize more advanced tools and is closely related to cardiac development, through dilated and hypertrophic cardiomyopathies.^1,9,15,20,21^

Regarding *CAV3* and HLHS relation, both cardiomyopathies have a strong morpho-histological and pathophysiological relation with HLHS, although its mechanisms have not yet been established since little is understood about the myocardial substrate in HLHS.^21,22,23,24,25,26,27^ The HLHS is divided into two types: slit-like (HLHS with mitral and aortic valves atresia) and peach-like (HLHS with stenosis in one or both of the valves or atresia in the other).^21^ For the peach type, there are enough reports associating HLHS with hypertrophy in the cavity of the diminutive left ventricle or in its septal.^27,28,29,30^ But the slit-like HLHS does not present sufficient material in the literature to support or deny any association between HLHS and cardiomyopathies. It is also possible that both types of HLHS could share similar morphological consequences, as demonstrated in the study of Rösner et al., where apicolateral hypertrophy of the hypoplastic left ventricle in the apical bulging HLHS phenotype was present in all anatomical subtypes.^28^ Additionally, fibroelastosis and dilated cardiomyopathy may be independent factors causing HLHS.^23,27,31^ Therefore, regardless of the cardiomyopathy (hypertrophic or dilated), it is recognized that intrinsic defects of left ventricular cardiomyocytes need to be evaluated as a cardiopathy-related genetic variant could affect the outcome in HLHS.^26,28,30,31,32^

In conclusion, the candidate genes for CHDs and HLHS highlighted in previous studies were not deleted in our patient’s case. According to the bioinformatic analysis, none of the genes in the deleted region is correlated with HLHS or CHDs, suggesting some other microdeletion in the patient genome, not identified in the karyotype. Taking into account the technical limitations of the karyotype performed on our patient, this may explain the lack of correlation with the literature. Based on that, an unidentified partial break in the gene *CAV3*, adjacent to the patient’s deletion, is hypothesized. Its association with cardiomyopathies and isolated cardiac defects is notorious, which, together with the literature reviewed, places it as a strong candidate for cardiac development problems, such as HLHS. It was impossible to carry out the confirmatory exam since the NB died. We emphasize the need to carry out high-resolution genetic tests in patients with HLHS to provide the information that can contribute to the confirmation or denial of this association.

Cardiac genes are still being investigated, so more candidate genes may appear as research in this area develops. The field of miRNAs, an important tool for the regulation of tissue gene expression, was not covered, and a separate study should be developed to elucidate these factors. Although there is no evidence of non-penetrance, it is also a possibility that should not be ruled out as it can be a complicating factor in the association of CHDs to a specific critical region.

## Data Availability

The database that originated the article is available with the corresponding author. CAAE: 74971917.2.0000.5683
